# Diseases of the Throat and Nose

**Published:** 1884-09

**Authors:** 


**Diseases of the Throat and Nose.** Including the Pharynx, Larynx, Trachea, Œsophagus, Nose and Naso-pharynx. By Morell Mackenzie, M. D., London. Volume II. Diseases of the Œsophagus, Nose and Naso-pharynx, with index of authors and formulæ for topical remedies. Illustrated. Philadelphia: P. Blakiston, Son & Co., 1,012 Walnut Street. 1884.

Dr. Mackenzie has been twelve years in completing this work, and it is a monument of his genius and ability in the special department of medical science to which he has devoted his life. He writes, in the preface: "Had I foreseen how much time and trouble the work would have cost me I should never have had the courage to undertake it."

The author devotes a chapter to the gullet, its anatomy, the instruments used in its examination, and the diseases to which it is subject; a chapter to the nose, its anatomy, rhinoscopy, nasal instruments, the diseases of these organs and the treatment; a chapter to "Diseases of the naso-pharynx and its diseases." The appendix gives special formulæ for topical remedies, etc.

We need not say that it is the most complete work published on diseases of the nose and throat; and every page bears the evidence of the distinguished ability which the author has devoted to its preparation. Those interested in this special class of diseases should possess such a work for study and reference.

